# Effects of intensive speech treatment on Mandarin speakers with Parkinson’s Disease: A review

**DOI:** 10.1097/MD.0000000000032900

**Published:** 2023-02-10

**Authors:** Qingqing Chen, Bailin Chen, Qin Wan, Yongli Wang, Jian Li, Zhaoming Huang

**Affiliations:** a Department of Education and Rehabilitation, Faculty of Education, East China Normal University, Shanghai, China; b KangDa College of Nanjing Medical University, Lian Yungang, China; c Faculty of Traditional Chinese Medicine, Naval Medical University (Second Military Medical University), Shanghai, China.

**Keywords:** effects, intensive speech treatment, Mandarin speakers, Parkinson’s Disease

## Abstract

Benefits of intensive speech treatment have been documented for a range of speech signs in English speakers with Parkinson’s Disease (PD). However, the answer to a critical question that whether the same treatment benefits speech variables including intelligibility in Mandarin speakers is still unclear. In order to develop a targeted speech treatment for Mandarin speakers with PD, we reviewed the efficacy of intensive speech treatment to improve vocal loudness and functional communication and discuss possible explanations for efficacy on Mandarin speakers with PD.

**Methods:**

Literatures about intensive speech treatment for Mandarin speakers with PD were retrieved from PubMed, Web of Science, Embase, China National Knowledge Infrastructure (CNKI), Wanfang and Weipu Database for Chinese Technical Periodicals (VIP) Database. Search strategy was (voice therapy OR speech therapy OR voice treatment OR speech treatment OR voice training OR speech training OR voice rehabilitation OR speech rehabilitation OR Lee Silverman voice treatment OR intensive speech treatment) and (Parkinson’s disease) and (Mandarin speakers OR Chinese OR Chinese people).

**Results:**

Five randomized controlled trials were selected and possible explanations for efficacy on individuals with PD are discussed. Further research directions are suggested.

**Conclusion:**

The existing evidence from treatment efficacy studies of intensive speech treatment provides support for improving vocal loudness, speech intelligibility, pitch and rate in Mandarin speakers with PD. Our future research will continue to work to conduct a large sample multicenter randomized controlled trial to provide high quality evidence and understand the basic mechanisms accompanying treatment-related change.

## 1. Introduction

Parkinson’s disease (PD) is now seen as a complex central neurodegenerative disease, which mostly occurs in middle-aged and elderly population. It is clinically characterized by tremor, bradykinesia, myotonia, and balance disorder. More than 6 million individuals worldwide currently diagnosed with the disease.^[1]^ Among these are many Mandarin speakers, as Mandarin is the most widely spoken native language in the world. More than 2,500,000 individuals in China have PD.^[2]^ Approximately 90% of individuals with PD develop the motor speech disorder of hypokinetic dysarthria, mainly including voice, articulation and prosody disorders^[3,4]^,which are characterized by reduced loudness, monotone, short phrases, inappropriate silences, short rushes of speech, harsh or breathy voice, imprecise consonants and increase/decrease/variable speech rate.^[5]^ As a result, reduced intelligibility, as well as consequent difficulties for communicative and social participation are exhibited among patients of PD, leading to a significant decline of their life quality.^[6]^

There is no consistent or significant effect between drugs and surgery (thalamotomy and pallidotomy) on the improvement of degenerating speech in patients with PD, and surgical treatment even has the risk of aggravating speech disorder. In recent years, researchers have shown great interest in deep brain high-frequency stimulation for the treatment of PD. However, with the development of clinical practice, Aldridge et al found that deep-brain stimulation (DBS) can worsen speech and make brain tissue irreversible changes in patients with PD.^[7]^ The efficacy of medical, surgical, and deep-brain stimulation treatments of dysarthria in Parkinson’s disease have been variable and generally disappointing. Therefore, the role of speech therapy in the process of speech rehabilitation has become increasingly prominent. Traditional speech therapy focuses on the subsystems of speech, including breathing, phonation, articulation, resonance, and improves the strength, coordination of movement, range of motion, and speed of articulation so as to enhance the overall speech function of patients. However, traditional speech therapy is mainly symptomatic, and has deficiencies such as short efficacy, poor persistence, and difficulty in generalization to daily communication.

Intensive speech treatment incorporates principles of motor learning and activity-oriented neuroplasticity,^[8]^ including intensive dosage (at least 16 times, 1-hour sessions over 1 month), high effort treatment, which may be beneficial for improving deficiencies in speech. Most of the studies performed on English speakers show benefits of intensive speech treatment across a range of speech variables, including objective acoustic measures of vocal loudness, articulation, intonation, and perceptual measures of voice quality and patients’ self-reported improvements in communicative effectiveness.^[9–13]^ To date, there is even fewer evidence to suggest intensive speech treatment can increase intelligibility in Japanese, Spanish, and German.^[14–16]^

Despite the large numbers of Mandarin speakers with hypokinetic dysarthria secondary to PD, however, whether the same treatment benefits speech variables including intelligibility in Mandarin speakers remains to be determined. Therefore, this review addresses the efficacy of intensive speech treatment to improve vocal loudness and functional communication in Mandarin speakers with PD. The underlying pathological mechanism of PD associated with vocal and speech changes is discussed, and explanations for efficacy of intensive speech treatment on individuals with hypokinetic dysarthria secondary to PD are reviewed, and further research is also proposed for Mandarin speakers with PD in China.

## 2. Possible physiopathology of parkinsonian dysarthria

Parkinson’s disease is generally believed to be caused by a loss of dopaminergic cells in the substantia nigra pars compacta of the basal ganglia.^[17]^ At present, the exact pathophysiological mechanism of speech and language disorder in this population is still unclear. Existing studies suggest that it may be related to the damage of cortical-basal ganglia thalamo-cortical circuit, motor executive dysfunction, auditory perception dysfunction and cognitive dysfunction.

### 2.1. Cortical-basal ganglia-thalamus-cortical circuit impairment

Speech production not only involves the classic Broca and Wernicke area, but also many other cortical areas and subcortical structures, such as primary motor cortex corresponding to the trunk and orofacial somatotopic areas,^[18]^ premotor area, supplementary motor area (SMA), prefrontal, thalamus, cerebellum, basal-ganglia and projective fibers between them, which increase the complexity of the function. Speech movements are initiated in the primary motor cortex corresponding to the trunk and orofacial somatotopic areas.^[19]^ The striatum is a high-level integrated structure that receives auditory and visual sensory information and conducts commands from the cerebrum. The efferent fibers of the striatum project from the globus pallidus to the thalamus, and then to the premotor area and orofacial primary motor cortex, forming a cortex-basal ganglia -thalamus-cortical circuit. Impairment of any part of this circuit may lead to speech disorder.

A study showed that the speech signs in patients with PD were related to the orofacial primary motor cortex, the recruitment changes of cerebellum, and the excessive involvement of the premotor area and prefrontal cortex.^[19]^ Similar to these findings, Liotto et al^[20]^ found the SMA and inferior lateral premotor cortex were hyperactivated during a speech production task in participants with PD. However, they pointed out that the small sample size (5 patients) and the absence of a control group made this finding preliminary. Another functional imaging study with positron emission tomography found that in PD there is an under activation in the right orofacial motor cortex and bilateral cerebellar hemispheres, abnormally increased regional cerebral blood flow in the right superior premotor cortex and bilateral dorsolateral prefrontal cortex, and overactivation of SMA.^[21]^

**Figure 1.**
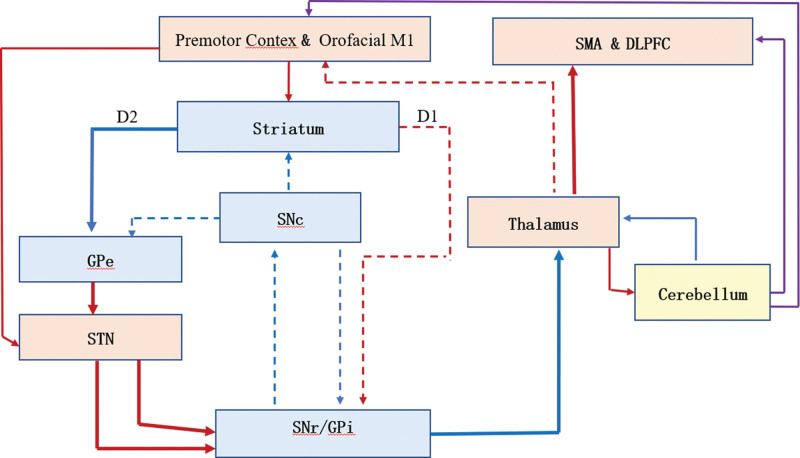
Cerebral activation abnormalities during speech in PD. Three main pathways through basal-ganglia, which accept and efferent information during speech production are: Cortex-Striatum-SNr/GPi-Thalamus-Cortex; Cortex-Striatum-Gpe-STN-SNr/GPi-Thalamus-Cortex; Cortex-STN-SNr/GPi-Thalamus-Cortex. Blue lines = inhibitory GABAergic projections, D1, D2 = D1 and D2 striatal dopamine receptors, DLPFC = The bilateral dorsolateral prefrontal cortex, dotted lines = underactivity of the projection, GPe = external globus palildus, GPi = internal globus pallidus, orofacial M1 = primary motor cortical areas, PM = premotor cortex, purple lines = corticocerebellar connection, red lines = excitatory glutamatergic projections, SNc = substantia nigra pars compacta, SNr = substantia nigra pars reticulata, STN = subthalamic nucleus, thick continued lines = hyperactive activities, thin continued lines = normal activities.

Marsden found in PD the frontal lobe cortical, basal ganglia and subcortical dopamine circuits are damaged, making the function of dopamine in the basal ganglia reduced, resulting in increased inhibitory output from the basal ganglia to the thalamus and brainstem motor areas, which causes speech disorders.^[22]^ The patients with thalamic lesions have reduced spontaneous speech, low vocal intensity, poor articulation clarity, and mild impairment of listening comprehension, reading and writing. This suggests that the hypothalamus may control speech output by connecting substantia nigra, reticular formation, and basal ganglia to premotor areas of the prefrontal cortex and orofacial primary motor cortical areas.^[23]^ A study by Moretti et al^[24]^ support that stimulation of the inferior colliculus nucleus can effectively activate the dorsolateral frontal cortex, which controlled speech output through cortex – basal ganglia – thalamus – cortical circuits. So far, there have been few functional imaging studies of dysarthria in PD, and it is still difficult to judge on the cerebral activation changes related to this abnormal changes (see Fig. 1).^[19]^

### 2.2. Speech motor executive function impairment

Speech movement is highly complex that requires the coordination of respiratory, phonation, articulation, resonance system and related nerves to accurately execute speech commands. In the speech motor regulation, the central nervous system extracts the information of neuromuscular expression corresponding to speech content from speech motor memory resources (the internal cue), and converts it into speech motor instructions, and which were finally executed through the vocalization motor system to produce speech (speech execution). Some research concluded patients with PD are unable to produce effective movement planning based on internal cues, resulting in impaired motor execution. In addition, patients with PD have bradykinesia, rigidity and tremor and respiratory disorders, which lead to incomplete glottis closure or asymmetry during vocalization,^[25]^ resulting in execution of accurate speech motor commands failed.

### 2.3. Auditory perceptual feedback system impairment

The loudness of speech in PD decreases with the progression of the disease, but patients are often unaware of their low vocal intensity. When prompted to speak loudly, they perceive themselves as “shouting.” But if they were given a recording of themselves and a recording of a normal person, they could discern the difference in loudness. This suggests that the hearing of this population is normal, but the processing of auditory perception is defective. Mollaei et al and Huang et al found that patients with PD will produce greater sound compensation when they listen to their own speech feedback, which has been artificially added with some interference.^[26,27]^ Moreover, after the interference sound, the sound intensity and fundamental frequency (F0) will also increase significantly,^[27]^ while normal people will not show these obvious changes. Further studies found when correcting the deviation of speech fundamental frequency feedback, the amplitude of cortical evoked potential P2 in PD patients is significantly higher than that in normal controls, and the brain electrical activity in the superior temporal gyrus, primary motor cortex, inferior frontal gyrus, and prefrontal cortex is significantly increased.^[28–30]^ These studies suggest people with PD lack awareness of their impaired loudness and have difficulties in appropriately adjusting loudness, which is possibly related to impaired auditory perception.

### 2.4. Cognitive function impairment

Language is one of the components of cognition process, and it is also the appearance of cognitive function. Progression of PD with time concomitant with aggravation of dysarthria suggests a link to the increasing severity of cerebral non-dopaminergic lesions.^[31]^ Studies have indicated that speech and language disorders in patients with PD are related to cognitive impairment, and with the decline of cognitive function, the speech and language function gradually deteriorates.^[32]^ The authors suggested that at least one other cognitive process (acoustic tone duration processing) accounted for the speech perceptual deficit in people with PD.^[33]^ A study about magnetic resonance imaging in patients with hypokinetic dysarthria from Liu^[34]^ found that the subcortical gray matter volume in the hippocampus of these patients is significantly reduced. In addition, Li et al^[35]^ also found that the brain electrical activity of cognition-related regions such as inferior frontal gyrus and prefrontal lobe increased significantly in patients with hypokinetic dysarthria. These studies may imply that the deficit in speech and language in PD is highly associated with cognitive decline. Therefore, screening of cognitive function is very important for the accurate clinical assessment of speech function in patients with PD.

## 3. Research outcomes on intensive speech treatment for Mandarin speakers with PD

**Figure 2.**
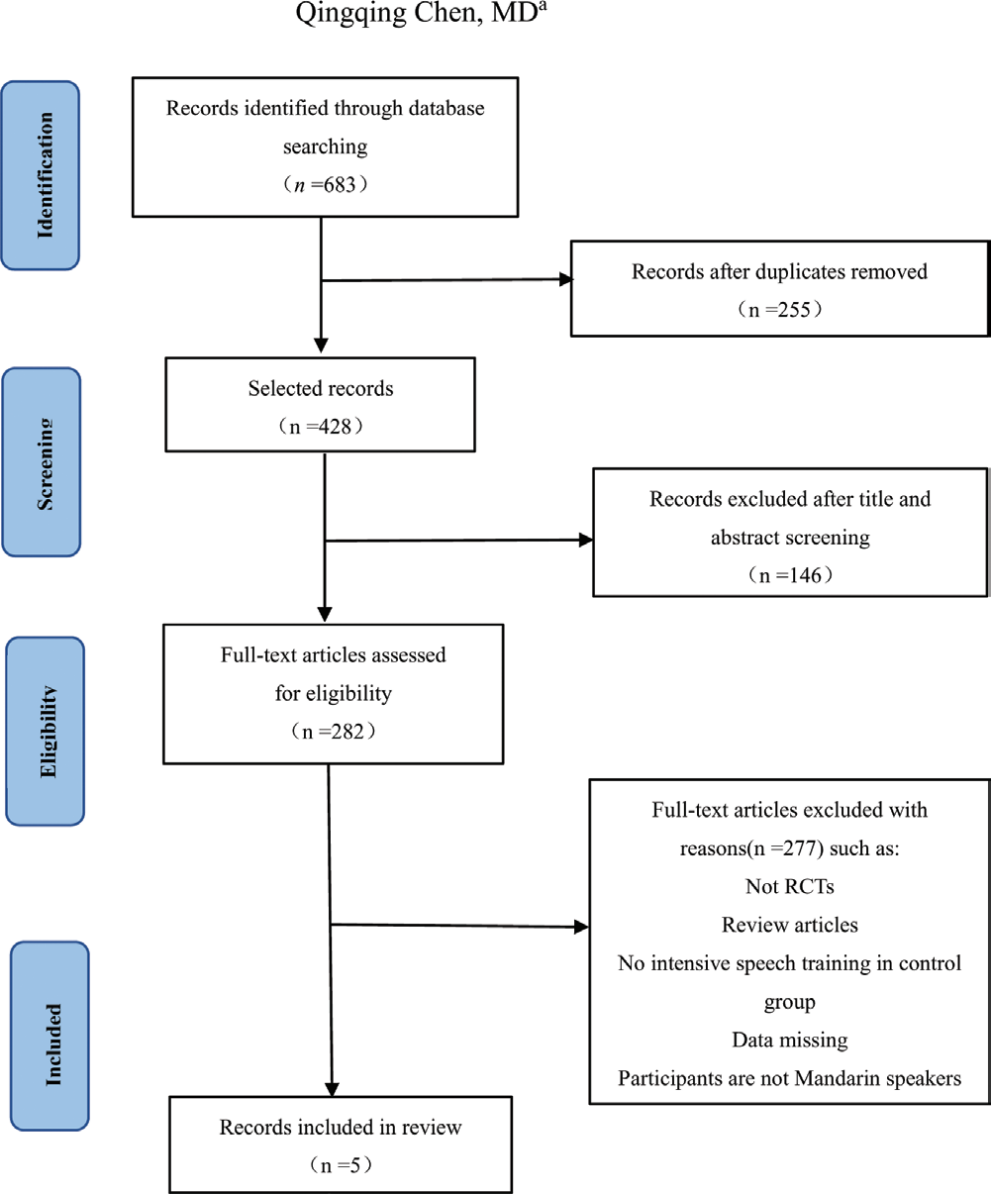
The flowchart of study selection process. RCTs = randomized controlled trials.

We searched 6 electronic databases to identify relevant studies from inception to December, 2022. Five randomized controlled trials (RCT) were included from 2010 to 2022 and are summarized in Table 1. The flowchart of study selection process is shown in Figure 2.

**Table 1:** Summary of RCT on intensive speech treatment for Mandarin speakers with PD.

Study (year)	Subject (N)	Age (mean (SD))	HY	PD duration	Intervention	Intensity	Evaluation index	Outcomes
Zhao et al^[36]^ (2021)	I = 42	I = 6.76 (1.60)	–	I = 5.12 (0.45)	I = IST + TST	I = 1 h/time, 3 times/wk, for 12 wk	VHI, WAB	I = Decreased VHI score and increased WAB score compared to control group (*P* < .05)
	C = 42	C = 61.72 (1.56)	–	C = 5.18 (0.42)	C = TST	C = 1 h/time,3 times/wk, for 12 wk		
Wu et al^[37]^ (2020)	I = 50	I = 48.76 (7.21)	–	I = 9.02 (3.71)	I = IST + TST	I = 30–40 min/time, 2 times/d, for 5wk	VHI, UPDRS-III, intelligibility	I = Decreased VHI, UPDRS-III score, improved intelligibility compared to control group (*P* < .05)
	C = 50	C = 48.75 (7.21)	–	C = 9.68 (4.11)	C = TST	C = 30-40 min/time,3times/d, for 3weeks		
Zhang et al^[38]^ (2022)	I = 36	I = 67.29 (3.60)	–	I = 5.00 (1.36)	I = IST + TST	I = 20–30 min/time, 2–3 times/d, for 3 mo	VHI, UPDRS-III, intelligibility	I = Decreased VHI, UPDRS-III score, improved intelligibility compared to control group (*P* < .05)
	C = 36	C = 66.93 (3.47)	–	C = 4.83 (1.14)	C = TST	C = 15–30 min/time, 2–3 times/d, for 3 mo		
Yang et al^[39]^ (2017)	I = 49	I = 66.83 (2.89)	I = 2.11 (0.27)	I = 5.12 (0.67)	I = IST + TST	I = 1 h/time, 4 times/wk, for 12 wk + homework (1 h/d on treatment day and 2 h/d on non-treatment days)	VHI, UPDRS-III, WAB, intelligibility	I = Decreased VHI, UPDRS-III and increased WAB, intelligibility compared to control group (*P* < .05)
	C = 49	C = 65.82 (2.56)	C = 2.09 (0.34)	C = 5.52 (0.16)	C = TST	C = 1 h/time, 4 times/wk, for 12 wk		
Tang et al^[40]^ (2016)	I = 32	I = 69.75 (4.35)	I = 2.02 (0.54)	I = 4.52 (3.26)	I = IST + TST	I = 1 h/time, 4 times/wk, for 12 wk + homework (1 h/d on treatment day and 2 h/d on non-treatment days)	VHI, UPDRS-III, WAB, intelligibility	I = Decreased VHI, UPDRS-III and increased WAB, intelligibility compared to control group (*P* < .05)
	C = 32	C = 68.45 (4.62)	C = 2.05 (0.48)	C = 4.64 (3.43)	C = TST	C = 40–60 min/time, 4 times/wk, for12 wk		

C = control, HY = Hoehn and Yahr scale, I = intervention, IST = intensive speech treatment, PD = Parkinson’s disease, RCTs = randomized controlled trials, TST = Traditional speech treatment.

The existing research data show subjective evaluation indicators such as speech intelligibility test, voice handicap Index, the West Aphasia Battery, and the unified Parkinson’s Disease Rating Scale-III were used to evaluate speech and communicative function after treatment in patients with PD. Although the sensitivity of subjective indicators is not strong, they can better reflect the actual communication ability and needs of patients. However, objective acoustic parameters are more reliable for detecting changes in voice. Therefore, the combination of subjective and objective indicators can more accurately reflect the speech function of patients in future studies.

The included RCTs show that speech intensive therapy benefits speech variables including intelligibility in Mandarin speakers and help them achieve long-term social communication by improving coordination and regulation of the speech subsystem.

## 4. Relevant evidence for efficacy of intensive speech treatment

In the sections above, we summarize the available RCTs for efficacy of intensive speech treatment on Mandarin speakers with PD. In this section, we review available evidence for efficacy of intensive speech treatment in further details.

### 4.1. Evidence for improvement on quality of voice

The low voice in patients with PD is often the first concern. The previous view suggested that reductions in vocal loudness are attributed partly to hypokinesia (reduced amplitude of movement) and rigidity. In recent years, abnormalities in central sensory processing (reduced perception of soft voice), internal cues (difficulty self-generating increased volume), and self-monitoring of speech output are now reported to contribute mainly.^[30,41,42]^ Fox and Ramig^[43]^ found vocal sound pressure levels (vocSLP) were 2 to 4 dB lower in PD patients than in age-matched, sex-matched healthy controls, corresponding to a 40% reduction in sensory volume. This finding suggests that PD patients have reduced voice frequency range, which also provides a basis for explaining their monotonous intonation and reduced prosody. A study by Li et al^[44]^ investigated the effect of intensive speech treatment on Mandarin speakers with PD. They found that voice loudness in monologue and reading was significantly increased in the treatment group, which increased the output of speech and promoted the effective communication in daily life. This result is consistent with other national studies. Raming et al^[45]^ found that participants treated with intensive speech treatment had significantly higher SPL than control groups and found no side effects for patients. This research indicated that with the increase of SLP, the degree of glottic closure of patients was improved, and the results were confirmed by later studies.^[46]^

Hoarseness and roughness are one of the speech characteristics of patients with PD, which not only affect the loudness of speech, but also lead to the decrease of speech intelligibility. Recent studies have found that the fundamental frequency (F0), harmonic noise ratio (HNR), Jitter and Shimmer are significantly correlated with the severity of PD.^[47]^ A study by Zhu et al^[48]^ found fundamental frequency, Jitter and Shimmer of the PD patients were higher than those of the normal people, and the HNR was lower than that of the normal people. Intensive speech treatment can relieve the stiffness of laryngeal, tongue and oral muscles, increase the degree of glottis closure, and improve the voice quality of PD patients. RCTs from Table 1 found that abnormal voice in Mandarin speakers with PD who undergo intensive speech treatment was improved and adverse impact on communication reduced.

A further study by Yang et al^[39]^ indicated patients with PD had some improvement in short breath, hoarseness, dry throat and vocal fold fatigue during speech, and with a more stable voice after intensive speech treatment. This is consistent with the results of previous studies.^[40]^ Li et al and Ramig et al reported that with the individuals with PD who undergo intensive speech treatment, the VHI score was reduced by 35.23%, irregular phonation, breathy voice, weak phonation, tense dysphonia were improved, the discomfort of the larynx was reduced, and the patient could communicate naturally in daily life with benign emotional reactions, and social participation capacity was gradually increased.^[44,45]^

### 4.2. Evidence for improvement on articulation function

Approximately 70% to 90% of patients with PD have articulation disorder which directly affects the understanding of the listener and reduces the patient’s communication ability and cause psychological disorders.^[49,50]^ Findings from a study by Shen, et al suggest patients with PD was manifested in the reduction of F2i/F2u in acoustic parameters,^[40]^ which was due to the fact that the resonance peak was related to the movement range of tongue, lip, pharynx, and mouth.^[51]^ However, the muscles of articulation organs were stiff and tremors with respiratory motor disorder in patients with PD. These abnormities cause flexibility of speech motor organs decreased, resulting in abnormal resonance peak in patients with PD. Another study by Novotny et al^[52]^ implied F2i/F2u had been significantly abnormal in patients with PD before they had obvious dysarthria. In terms of vowel articulation, shorter maximum phonation time, shorter formant duration and slower vocalization are the main characteristics in patients with PD. It is considered that the inaccuracy of consonant may also be one of the most powerful indicators of speech disorder in PD.^[52]^ Intensive speech treatment has a good clinical effect on improving the articulation movement. Sapir^[41]^ researched the effect of intensive articulation therapy on improving the articulation movement of PD patients and found that F2i/F2u had significant changes after treatment, indicating that tongue movement flexibility had improved. Another study by Cannito et al^[53]^ demonstrated a significant improvement in speech intelligibility after undergoing intensive speech treatment, and this improvement still lasted for 6 months after treatment. A case report by Yang et al^[54]^ found the formant concentration ratio was decreased after treatment, while the vowel space area and vowel articulation index were increased. Another study by Baumann et al^[15]^ found that intensive speech treatment showed a positive impact on articulation accuracy, and moreover, functional magnetic resonance imaging showed that the cortical activation in the right superior temporal gyrus was significantly increased, indicating that the improvement of articulation accuracy was related to the degree of cortical activation in the right superior temporal gyrus in patients with PD.

### 4.3. Evidence for improvement on speech prosody

It has been reported that 20% of patients with PD have prosodic disorder in speech, such as syllables being repeated, shortened or prolonged, inappropriate pauses, monotone, lack of stress and emotional changes.^[55]^ A few studies found participants with PD also showed changes in speech rate and pause compared to a healthy group.^[56,57]^ The standard deviation of speech fundamental frequency (F0SD) is significantly correlated with the severity of PD.^[51]^ These results indicated with the progression of PD, the speech prosody gradually deteriorates.

A study in 2001 by Baumgartner et al^[13]^ found patients with PD that were treated with intensive speech treatment not only improved the hoarseness, but also monotone. A RCT by Tang et al and Yang et al confirmed that patients treated with intensive speech treatment had significantly improved shortness of breath and pauses when speaking, longer sentences were produced and loudness was significantly increased. Even communicating in a noisy environment, they can be more easily heard.^[40,41]^ Whitehill et al^[58]^ who investigated the effect of intensive speech treatment on Cantonese speakers with PD found that although there was no significant difference in the accuracy of single word intonation, the sentence intonation was significantly improved compared with that before treatment. Similar to these findings, a RCT by Yang et al^[39]^ found that speech repetition and fluency of participants with PD were improved after intensive speech treatment. A case report by Yang et al^[54]^ found that slowed speech rate and short rushes of speech were improved significantly in individual with PD. Explanation for efficacy on speech prosody is possibly that in the course of the treatment, the patient is allowed to continue to pronounce vowels “Ah,” which is to take into account the breathing control training at the same time, so as to achieve the respiratory support capacity.

## 5. Explanations for efficacy of intensive speech treatment

Intensive speech treatment addresses sensory kinesthetic and internal cueing deficits, flexibility of speech motor organs in patients with PD through intensive voice, intensive articulation and intensive respiration. High effort mode of treatment delivery is unique, which is consistent with theories of motor learning and principles that drive activity-dependent neuroplasticity, both of which may be important for long-term carryover and generalization of improved vocal loudness to functional communication outside of the clinical setting and maintain the changes over time.^[59]^ Repetitive, simple, high-intensity training can help people with PD relearn a new internal cue for the amount of effort they need to produce for normal loudness, accurate articulation and normal breath, then which are recalibrated to their sensory perception to recognize them are within normal limits.^[60]^ Meanwhile, during treatment, the patient is allowed to continue to pronounce vowels “Ah,” which is to take into account the breathing control training at the same time. In addition, high intensive training increases amplitude of motor output and coordinates effort across multiple speech production subsystems that may be impaired, which contribute to relieve the stiffness of muscles in laryngeal, tongue and mouth, facilitating degree of glottic closure.

## 6. Conclusion and directions for future studies

The pathophysiology of PD dysarthria is complex and, at least in part, different from that of limb dysfunction. Aiming at the possible pathological mechanism of speech disorder in PD, the cumulative evidence from treatment efficacy studies of intensive speech treatment provides support for improving vocal loudness, speech intelligibility, pitch, rate in English speakers with PD. However, until recently, there has been little empirical evidence to support this efficacy on Mandarin speakers with PD in China. In this paper, we have reviewed the available evidence on this issue and possible explanations for efficacy of intensive speech treatment. A few studies have provided empirical support for efficacy of intensive speech treatment on Mandarin speakers with PD. However, whether the underlying mechanism is suitable for Mandarin speaking PD patients still deserves further study. And physiologically based hypotheses to explain the speech disorders in individuals with PD are limited to date. Therefore, our future research will continue to conduct a large sample multicenter randomized controlled trial to provide high quality evidence on efficacy of intensive speech treatment in Mandarin speakers with PD and understand the basic mechanisms accompanying treatment-related change.

## Author contributions

**Conceptualization:** Qingqing Chen, Qin Wan, Zhaoming Huang.

Data curation: Yongli Wang.

Funding acquisition: Zhaoming Huang.

Methodology: Jian Li.

Project administration: Qingqing Chen, Bailin Chen, Qin Wan, Yongli Wang, Zhaoming Huang.

Writing – original draft: Qingqing Chen.

Writing – review and editing: Qingqing Chen, Zhaoming Huang.
